# Constructing the suprachiasmatic nucleus: a watchmaker's perspective on the central clockworks

**DOI:** 10.3389/fnsys.2015.00074

**Published:** 2015-05-08

**Authors:** Joseph L. Bedont, Seth Blackshaw

**Affiliations:** ^1^Department of Neuroscience, Johns Hopkins University School of MedicineBaltimore, MD, USA; ^2^Department of Ophthalmology, Johns Hopkins University School of MedicineBaltimore, MD, USA; ^3^Department of Physiology, Johns Hopkins University School of MedicineBaltimore, MD, USA; ^4^Department of Neurology, Johns Hopkins University School of MedicineBaltimore, MD, USA; ^5^Center for High-Throughput Biology, Johns Hopkins University School of MedicineBaltimore, MD, USA

**Keywords:** circadian, suprachiasmatic, developmental neuroscience, cell fate, transcription factors, clock genes, chronotype, axon pathfinding

## Abstract

The circadian system constrains an organism's palette of behaviors to portions of the solar day appropriate to its ecological niche. The central light-entrained clock in the suprachiasmatic nucleus (SCN) of the mammalian circadian system has evolved a complex network of interdependent signaling mechanisms linking multiple distinct oscillators to serve this crucial function. However, studies of the mechanisms controlling SCN development have greatly lagged behind our understanding of its physiological functions. We review advances in the understanding of adult SCN function, what has been described about SCN development to date, and the potential of both current and future studies of SCN development to yield important insights into master clock function, dysfunction, and evolution.

## From sundials to watches: the evolution of the central clock

From cyanobacteria to humans, a circadian system restricting behavior to specific intervals in the solar day is present. Its ubiquity testifies to the system's utility, allowing organisms to maximize energy efficiency while minimizing environmental risks variant within distinct niche-specific ranges over the solar cycle. In mammals, circadian rhythms regulate crucial homeostatic processes including feeding, metabolism, sleep/wake, hormone secretion, body temperature, waste elimination, and arousal.

In most life forms, molecular clocks control the circadian system on a cellular level. Generally, this takes the form of a negative feedback loop, with a heterodimeric transcriptional complex (in mammals, Bmal1/Clock) driving the expression of genes which feed back to inhibit their own transcription (in mammals, Per and Cry genes). Various sequestration and degradation mechanisms can delay or accelerate this inhibition, letting environmental cues such as food availability, exercise, external temperature, and most importantly light modify the molecular clock's phase and period (reviewed in Buhr and Takahashi, 2013; Yamanaka et al., 2013; Refinetti, 2015).

However, as multicellular organisms arose, some cells became isolated from the primary circadian zeitgeber, or “time-giver”: the sun. In response, specialization began. Many nervous system subdivisions were still accessible to light, and remained photosensitive. In non-mammalian species, this included not just retina, but also the pineal gland (Kappers, 1981; Cahill, 1996) and other deep-brain photoreceptors (von Frisch, 1911; Hartwig and van Venn, 1979; Moore and Whitmore, 2014). The clockworks in these tissues sometimes became synchronous, self-sustaining oscillators, and began to utilize both synaptic and humoral signals such as melatonin to communicate solar time to tissues cut off from direct light input (Steele et al., 2003, 2006; Noche et al., 2011; reviewed in Underwood et al., 2001). In many species, some of these self-sustaining oscillators are essential alone or in combination to coordinate behavioral rhythms. For instance, the eyes are necessary for behavioral rhythmicity in quail, the pineal is essential in many species of sparrows, and both play important roles in pigeons (reviewed in Underwood et al., 2001).

However, in mammals, all of these tissues except the retina lost their photosensitivity, and self-sustaining oscillators in most brain regions outside the suprachiasmatic nucleus (SCN) are short-lived or absent entirely, with a few notable exceptions such as the olfactory bulb and retina (Tosini and Menaker, 1996; Abe et al., 2002; Granados-Fuentes et al., 2004). To fill this void, the SCN evolved to become the mammalian brain's master light-entrained clock. This required a substantial change in the region's gene expression and function; for example, the SCN of lower vertebrates such as frogs express a somewhat different suite of neurotransmitters than in mammals, and the amphibian SCN appears to modulate light-dependent control of skin pigmentation, an ability that it lacks in higher vertebrates (Tuinhof et al., 1994a; Abrahamson and Moore, 2001; Dulcis and Spitzer, 2008). In birds, meanwhile, the medial and/or visual SCN in many ways shares more anatomical and functional characteristics with the mammalian SCN than does the amphibian SCN (reviewed in Underwood et al., 2001; Yoshimura et al., 2001; Cantwell and Cassone, 2006a,b; Ikegami et al., 2009). However, the avian central circadian system remains more distributed, with the retina and pineal playing more important roles in light-entrained circadian function than the medial and visual SCN in many species (reviewed in Underwood et al., 2001; Steele et al., 2003).

In contrast, in mammals the SCN is absolutely essential for behavioral rhythmicity. It has by far the most robust molecular clockwork in the brain (Abe et al., 2002), which is essential for many free-running behavioral rhythms in the absence of environmental light cues (Moore and Eichler, 1972; Stephan and Zucker, 1972; Husse et al., 2014). The SCN is also clock-independently required for entrainment of circadian behavior by light (Moore and Eichler, 1972; Stephan and Zucker, 1972; Husse et al., 2014). Thus, both master pacemaker function and photoentrainment appear to be centralized in the SCN of mammals. Though this evolutionary innovation not shared with other vertebrates, the marked preservation of many of its features across all mammals studied to date suggests that it has been well conserved within our clade (Cassone et al., 1988).

To fulfill its central role in controlling circadian rhythms, the mammalian SCN uses a complex, interdependent hierarchy of signaling molecules to integrate information from assorted afferents and the multiple, robust clocks located in its subdivisions. This diversity implies complicated developmental control of heterogeneous cell fate decisions, formation of assorted connections, and ontogeny of the central clockworks during SCN development. The remainder of this review will focus on the developmental and, to a lesser extent, evolutionary mechanisms that underpin these various aspects of the master clockworks.

## Forging the gears: specification and differentiation in the SCN

### Neurotransmitter diversity in the adult SCN

In developmental neuroscience, a given neuron's suite of neurotransmitters is often used as a convenient proxy for cell fate. Highlighting its complexity, the SCN secretes literally hundreds of distinct neuropeptides derived from at least 24 prohormones and numerous other proteins (Southey et al., 2014), along with cytokines, small-molecule neurotransmitters like GABA and nitric oxide, and others. Many of these signals have well-understood circadian roles (reviewed in Slat et al., 2013). For example, vasoactive intestinal peptide (Vip) and gastrin releasing peptide (Grp) maintain synchrony of SCN cellular oscillators and behavioral rhythmicity, and entrain them to light (Piggins et al., 1995; Harmar et al., 2002; Colwell et al., 2003; Aton et al., 2005; Brown et al., 2005; Gamble et al., 2007; An et al., 2011; Maywood et al., 2011). Arginine vasopressin (Avp) also strengthens SCN and behavioral circadian rhythmicity, but unlike Vip and Grp, potentiated phase shifting in animals lacking its receptors suggests Avp signaling acts to buffer and restrain light entrainment of the central clock (Li et al., 2009; Maywood et al., 2011; Yamaguchi et al., 2013). Prokineticin-2 (Prok2) acts primarily as an output that couples the SCN to other brain regions (Cheng et al., 2002; Prosser et al., 2007). More recently, high-throughput peptidomic and circuit mapping approaches have identified novel SCN signals that play important parts in circadian physiology, such as integration of glutamatergic SCN input by the neuropeptide little SAAS and the as-yet-unidentified cue(s) secreted by neuromedin S-(+) neurons that are essential for SCN pacemaker function (Atkins et al., 2010: Lee et al., 2015).

But while our knowledge regarding individual SCN signals continues to swell at an accelerating rate, their interactions are less well-documented, despite the importance of synergy among them for SCN circadian function. For instance, both Grp and Avp partially compensate for loss of Vip's synchronizing effects in slice culture (Brown et al., 2005; Maywood et al., 2011). Furthermore, cannulated neuropeptides have strikingly different effects *in vivo* when the SCN neuropeptide network is compromised. Under these conditions a single Grp pulse, which normally does not disrupt SCN or behavioral rhythm consolidation even at high doses (Piggins et al., 1995; An et al., 2013), deconsolidates activity rhythms (Bedont et al., 2014). Meanwhile, a Prok2 pulse that normally has negligible circadian effects (Cheng et al., 2002) massively shifts both phase and period of activity rhythms when the network is disrupted (Bedont et al., 2014). The latter may reflect an ancestral function of Prok2 normally overridden by the mammalian SCN network; in crayfish hematopoietic system, the prokineticin-like protein astakine-2 has a surprisingly similar effect on circadian period, though almost surely through a very different mechanism from Prok2 (Negri et al., 2005; Watthanasurorot et al., 2013).

**Figure 1:**
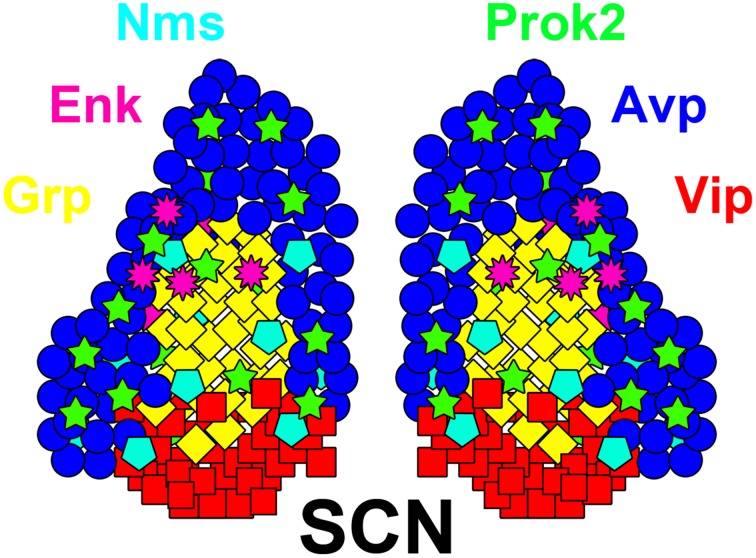
**Neuron diversity in the adult SCN**. A diagram of the adult SCN, showing a subset of its many neuropeptidergic populations. Neuropeptide colors are indicated by their names, positioned around the SCN. Note that many of these neuropeptides cross or exist outside of the classical core and shell SCN subdomains. Also note that many of these neurotransmitters are at least partially co-expressed, such as in Vip/Grp neurons, though the various probable combinations are not shown here for simplicity. Adapted from Bedont et al. (2014) Graphical Abstract.

Classically, the panoply of different SCN cell types were neatly divided between a Vip(+) ventrolateral (vlSCN) core and an Avp(+) dorsomedial (dmSCN) shell (Abrahamson and Moore, 2001); however, additional domains such as the central SCN exist in many mammals, and other neuropeptidergic cell types cross domain boundaries (Figure 1; reviewed in Morin et al., 1992; LeSauter and Silver, 1999; Abrahamson and Moore, 2001; Morin, 2007; Bedont et al., 2014; Allen Brain Atlas^1^). Many cells also express multiple signals, like mouse and rat Vip/Grp(+) SCN neurons, and mouse Vip/Nms-(+) and Avp/Nms-(+) neurons (Okamura et al., 1986; Albers et al., 1991; Lee et al., 2015). There is also extensive patterning of non-neuropeptide SCN gene expression, often along non-core/shell axes. For example, many neuropeptide receptors appear to be expressed in gradients along the anterior/posterior axis in mouse SCN, with Vipr2 and Prokr2 highest in the anterior shell and Avpr1a and Avpr1b highest in the posterior shell (An et al., 2012; Allen Brain Atlas). Thus, though the neuropeptides Vip, Grp, Avp, and Prok2 will be referred to frequently in the following subsections, it should be understood that these neuropeptides almost certainly do not delineate all key neuronal subtypes in the SCN.

### Patterning, specification, and neurogenesis in the nascent SCN

**Figure 2:**
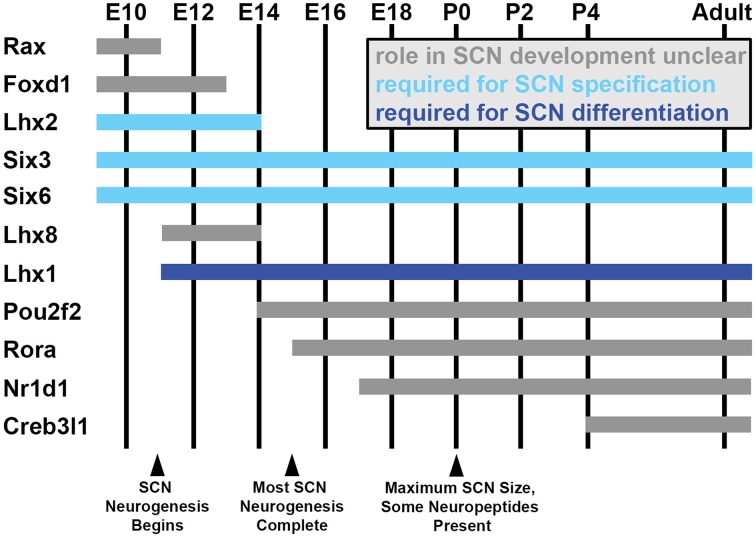
**SCN transcription factor expression during development**. Estimated ages of expression for a subset of hypothalamus- and SCN-enriched transcription factors in the developing mouse SCN between embryonic day (E) 10 and adulthood, selected based on interesting expression patterns and/or known developmental functions (indicated by bar color). Note that many general hypothalamic transcription factors (expressed prior to E10) are downregulated as the transcriptional network controlling SCN development ramps up. Not shown in this figure, other transcription factors expressed throughout the SCN during its development in turn become progressively compartmentalized to specific subdomains as the SCN matures (ex: Lhx1, Rorα).

As in much of the rest of the hypothalamus, the study of SCN development has badly lagged understanding of adult SCN diversity, though recent studies have begun to shed light on the topic (reviewed in Bedont et al., 2015). Broadly, the developing anterior hypothalamus (AH) begins as a unitary developmental unit that subsequently divides into two developmentally distinct compartments: a ventral subdivision (vAH) that gives rise to nuclei of the circadian and sleep systems, including the SCN, and a dorsolateral subdivision (dlAH) that gives rise to nuclei of the neuroendocrine system. Very early SCN development shares much in common with the rest of the AH, and later the vAH. For instance, Sonic hedgehog (Shh) signaling from the nearby basal plate is essential for its early patterning and initial specification (Shimogori et al., 2010). Many general AH and vAH transcription factors such as *Rax, Foxd1, Nkx2.2, Lhx2, Six3*, and *Six6* are also expressed in the vAH neuroepithelium prior to the onset of SCN neurogenesis, though expression of many of these factors is lost as the SCN becomes more differentiated from surrounding tissues (Figure 2; Shimogori et al., 2010; VanDunk et al., 2011; Pak et al., 2014; Allen Brain Atlas). Nonetheless, their transient expression is important for early specification of the SCN lineage.

For instance, *Lhx2*^−/−^ mice fail to down-regulate dlAH markers such as *Sim1* and *Otp* and up-regulate early vAH gene expression such as *Vax1* and *Lhx1* by embryonic day (E) 12.5, when AH subdivisions are normally clearly defined (Roy et al., 2013). These mice would almost certainly fail to specify an SCN (Figure 2). However, *Lhx2* is expressed in cells surrounding the SCN by birth, rather than in the SCN proper, suggesting it may play more of a role in early specification of dlAH and vAH identity than in promoting SCN specification *per se* (VanDunk et al., 2011).

Unlike *Lhx2* and many other early AH and vAH factors that are down-regulated in SCN later in development, *Six3* expression remains expressed in the vAH throughout the lifespan, and is required cell-autonomously for initial SCN specification (Figure 2; Shimogori et al., 2010; VanDunk et al., 2011). Its homolog *Six6* has a similar, although more restricted, enduring vAH expression pattern, and is also required for initial SCN specification (Figure 2; Shimogori et al., 2010; Clark et al., 2013). Robust but transient *Fzd5* receptor expression in mitotic SCN neuroepithelium also suggests that Wnt signaling may regulate SCN specification and/or neurogenesis, but this possibility has so far not been addressed (Shimogori et al., 2010; VanDunk et al., 2011).

**Figure 3:**
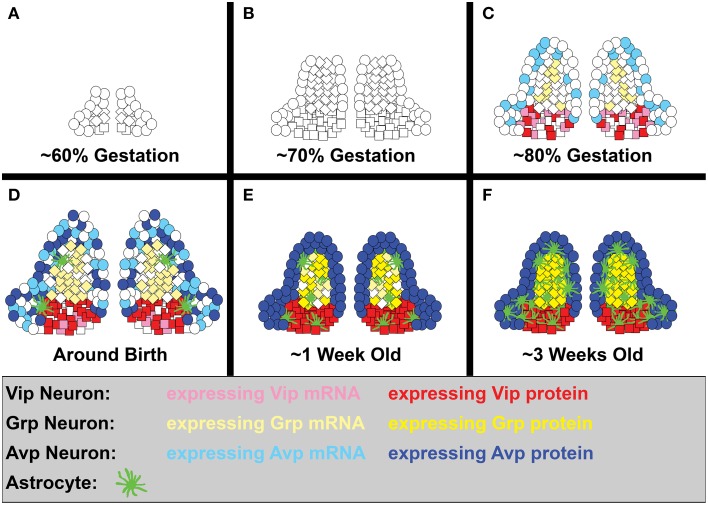
**SCN neurogenesis and differentiation**. A simplified diagram showing the development of Vip, Grp, and Avp neurons, as well as astrocytes, in the rodent SCN. While domain boundaries are depicted as being relatively static here to aid in illustrating our points, note that they are actually quite dynamic over the course of development. **(A)** Early SCN neurogenesis begins at ~60% of gestation (hamster E9.5, mouse E12, and rat E13.5). **(B)** By ~70% of gestation (hamster E11, mouse E13.5, rat E15), most ventrolateral neurons have already been produced, while dorsomedial neurogenesis is still in full swing. **(C)** The final major burst of SCN neurogenesis (including a number of ventrolateral neurons) occurs at ~80% of gestation (hamster E12.5, mouse E15, rat E17). Shortly thereafter, many SCN neuropeptide transcripts, but only Vip protein, are detectable (hamster E13, mouse E18-P0). **(D)** Shortly before and after birth, the first SCN astroglia appear (hamster E15, rat E20), Avp protein expression becomes detectable (P1), and SCN neuron number peaks (hamster P2). **(E)** By roughly a week into life, a major jump in astrocyte number has occurred (rat P3-P4), cell death brings neuron numbers down to adult levels (hamster P5, mouse P7, rat P6), and Grp protein expression is detectable (hamster P8). **(F)** By the end of the second major jump in astrocyte number (rat P20-25), the SCN is largely mature. At this stage, the fate of its neurons and the subdomain boundaries they form are relatively stable, though they may remain somewhat malleable under circadian challenge from the environment.

The bulk of SCN neurogenesis occurs between ~60–80% of gestation in rodents, at approximately E9.5-12.5 in hamster, E12-15 in mice, and E13.5-17 in rat (Figures 3A–C; Altman and Bayer, 1978; Crossland and Uchwat, 1982; Davis et al., 1990; Antle et al., 2005; Kabrita and Davis, 2008). Studies in mouse and hamster suggest that genesis of vlSCN neurons consistently peaks earlier than dmSCN neurons in rodents, in contrast to the anteroposterior timing of SCN neurogenesis, which shows much greater species-dependent variation (Davis et al., 1990; Kabrita and Davis, 2008). Correspondingly, cell types enriched in hamster SCN core and central domains such as Vip, Grp, and calbindin (Calb1) are mostly born early, while Avp(+) neurons of the shell can be born at any time during the period of neurogenesis (Antle et al., 2005) (Figures 3A–C). An interesting exception to this trend is a distinct subset of Vip and Calb1 neurons that are born at the end of rat SCN neurogenesis (Abizaid et al., 2004), likely corresponding to the final burst of vlSCN neurogenesis in hamsters (Figures 3C,D; Davis et al., 1990). As in most brain regions, astrogliogenesis follows neurogenesis in the SCN, with the astrocyte marker glial fibrillary acidic protein (Gfap) first detectable shortly before birth, at E15 in hamsters and E20 in rats (Figure 3D; Botchkina and Morin, 1995; Munekawa et al., 2000).

### Control of neuronal differentiation in the developing SCN

The mechanism restricting generation of individual SCN cell types to discrete temporal windows is unclear. Given that SCN neuron number peaks at ~P2 in hamsters (Figure 3D; Muller and Torrealba, 1998), there is likely a several-day maturation window for SCN neurons. Thus, one possibility is that extracellular cues could differentially influence the fate of SCN neurons born at different times. For example, although cells of the SCN do not themselves express *Shh*, at early stages of hypothalamic neurogenesis they are immediately adjacent to cells that do (Shimogori et al., 2010; Alvarez-Bolado et al., 2012). By E14 in the mouse, however, *Shh* expression is downregulated and moved further posterior from the SCN. Late-born SCN cells are thus exposed to lower Shh levels than early-born cells, possibly influencing their fates (Kabrita and Davis, 2008; Shimogori et al., 2010). Any changes in exposure to Wnt family members during this time would also likely affect *Fzd5*(+) SCN progenitors (VanDunk et al., 2011). There may even be secreted signals from the maturing SCN itself that influence the fates assumed by late-born neurons (see below). Conversely, SCN progenitor competence to respond to external cues may also change over the course of neurogenesis.

Differential expression of cell-autonomous factors may also play a direct role in birth-date biasing of SCN cell fate, but so far the only known transcription factor involved in any facet of SCN differentiation is LIM homeodomain transcription factor 1 (Lhx1), the earliest selective marker of developing SCN (Figure 2; Shimogori et al., 2010; VanDunk et al., 2011). In *Six3-Cre/+;Lhx1^lox/lox^* mice, in which Lhx1 function is disrupted beginning early in hypothalamic neurogenesis, the SCN fails to terminally differentiate (Bedont et al., 2014). This leads to reductions in several neuropeptide lineages important for circadian function, including neurons expressing *Vip, Grp, Avp*, and *Prok2*, among others (Bedont et al., 2014). Both bioinformatic and luciferase analysis suggest that Lhx1 directly regulates expression of some of these neuropeptides in the SCN (Bedont et al., 2014; Hatori et al., 2014). Interestingly, induction of *Lhx1* expression coincides with the beginning of SCN neurogenesis in mouse (Kabrita and Davis, 2008; Shimogori et al., 2010; VanDunk et al., 2011), and the mostly earlier-born Vip and Grp neurons are more severely depleted than Avp neurons in the *Lhx1*-deficient SCN (Bedont et al., 2014).

However, it seems unlikely that Lhx1 is necessary for early cell fate decisions or regional patterning in the SCN. Lineage-specific sub-domain markers expressed prior to E16.5 so far seem to be only modestly affected or completely unaffected in *Six3-Cre/+;Lhx1^lox/lox^* SCN, and morphological defects in SCN shape and neuron number first appear postnatally in mutant mice (Bedont et al., 2014). Histological data also suggests these morphological defects are shared by *Rorα^Cre/Cre^;Lhx1^lox/lox^* SCN (though this was not commented on in the original publication), and the Vip lineage is also depleted in this line, which is predicted to delete *Lhx1* in SCN beginning at E15, after the generation of Vip(+) neurons is mostly complete (Hatori et al., 2014). While Vip loss appears less severe in *Rorα^Cre/Cre^;Lhx1^lox/lox^* than *Six3-Cre/+;Lhx1^lox/lox^* SCN, interpretation of this phenotype is confounded by the substantially less efficient *Lhx1* deletion seen with the *Rorα^Cre/Cre^* driver. It thus seems likely that the less severe phenotypes seen in *Rorα^Cre/Cre^;Lhx1^lox/lox^* animals result primarily from less efficient *Lhx1* deletion, rather than deletion of *Lhx1* later in development (Bedont et al., 2014; Hatori et al., 2014). Thus, the sum of these data suggest that Lhx1 primarily acts downstream of other genes that control initial specification of SCN identity.

Instead, Lhx1 likely acts as one of several essential cogs in a complicated transcriptional network controlling SCN terminal differentiation. One probable downstream component is downregulated in *Rorα^Cre/Cre^;Lhx1^lox/lox^* SCN: the transcription factor *Creb3l1* (Hatori et al., 2014). Creb3l1 is a potent, direct regulator of *Avp* expression, whose circadian expression tracks *Avp* in adult SCN (Greenwood et al., 2014). Conversely, unlike *Vip*, both *Avp* and *Creb3l1* lack predicted Lhx1 binding sites in their proximal promoters (Bedont et al., 2014). Thus, Lhx1 likely indirectly controls expression of *Creb3l1* and in turn *Avp*, rather than directly controlling expression as it does for *Vip* (Hatori et al., 2014).

Interestingly, much like the less severe loss of *Avp* in the *Six3-Cre/+;Lhx1^lox/lox^* SCN relative to *Vip*, the reduction in *Creb3l1* expression in the *Rorα^Cre/Cre^;Lhx1^lox/lox^* SCN was less severe than for *Vip* (Bedont et al., 2014; Hatori et al., 2014). Allen Brain Atlas data suggests that *Creb3l1* induction may lag behind the earliest *Avp* expression observed in the SCN during development (Figures 2, 3D). Thus, Creb3l1 may play a more important role in differentiation or maturation of only later-born subpopulations of *Avp* neurons. Alternatively, it could be required for maintenance, but not induction, of *Avp* expression. Directly testing Creb3l1 function in the developing SCN is a promising direction going forward, as is testing the contribution of other potential members of the transcriptional network to which Lhx1 and Creb3l1 belong. Candidate factors including Lhx8, Rorα, Rorβ, and Pou2f2, among many others, have been identified as developmentally regulated SCN transcripts by recent screening efforts, but their roles in SCN development have not been examined (or, in the case of Rorα, not examined in detail) (Figure 2; Shimogori et al., 2010; VanDunk et al., 2011).

*Lhx1* is also an early, robust SCN marker in amphibians (Dulcis and Spitzer, 2008), despite key differences in gene expression with mammals, such as prominent neuropeptide Y (Npy) expression in frog SCN neurons (Tuinhof et al., 1994b). Frog SCN neurons also express an *Lhx5* homolog, which is absent in mammalian SCN but co-expressed with *Lhx1* in the mammalian spine, where the two transcription factors jointly regulate terminal differentiation of neuropeptide lineages including Npy(+) cells (Brohl et al., 2008; Shimogori et al., 2010; Allen Brain Atlas). Thus, it would be interesting to assess whether *Lhx5* has become re-activated in the human SCN during evolution, as to our knowledge, our species is unique among mammals in expressing Npy locally there (Moore, 1989). Given such tantalizing hints, we suspect that changes in the regional transcriptional network are a major proximal cause of evolutionary changes in the SCN.

### Maturation and cell fate plasticity in the developing SCN

The neurotransmitter fate(s) chosen by SCN neurons begin to become clear late in embryonic development. Many SCN neuropeptide mRNAs, including *Vip, Avp, Grp*, and *Prok2*, are first detectable by *in situ* hybridization between E18 and birth in mouse SCN, though generally in numbers lower than seen in adults, suggesting that not all SCN neurons are differentiated at this time (Figure 3C; Shimogori et al., 2010; VanDunk et al., 2011; Bedont et al., 2014; Allen Brain Atlas). In contrast, SCN neuropeptide proteins first become detectable at more variable ages than do their mRNAs. In hamster SCN, Vip is present by E13 (only slightly earlier than *Vip* mRNA in mouse, adjusted for gestation), while Avp is present by P1, Grp is present by P8, and substance P (Sp) is present by P10 (Figures 3C–E; Romero and Silver, 1990; Botchkina and Morin, 1995; Antle et al., 2005). The first major increase in rat SCN Gfap expression, indicating astrocytic maturation, also occurs during this time at ~P3-P4 (Figure 3E; Munekawa et al., 2000).

Staggered neuropeptide availability may have profound implications for SCN maturation. The earliest synapses form in rat SCN at E19, shortly after Vip first appears in the E13 hamster SCN adjusted for gestation (Moore and Bernstein, 1989). Hamster Vip neurons already begin to extend axons as early as E15, though the scarcity of synapses suggests that any signaling they engage in during neonatal life is likely paracrine (Botchkina and Morin, 1995). This situation changes rapidly with increasing synapse formation between P2-P6, shortly after Avp expression is first detected, followed shortly thereafter by another increase that drives synapse density to adult levels by P10 (Moore and Bernstein, 1989).

SCN cell death begins as synapse formation increases, with substantial death between E21-P6 in rats and P1-P7 in mice, and a sharp drop in neuron number between P2-P5 in hamster (Figures 3D,E; Moore and Bernstein, 1989; Muller and Torrealba, 1998; Ahern et al., 2013). Dying neurons are isolated from neuronal clusters that form during this time in rat SCN, hinting that cell-cell communication regulates SCN neuron survival (Moore and Bernstein, 1989).

It isn't until around this time that SCN adult-like subdomains start to become distinguishable. New transient populations become apparent in the SCN even after birth, with a dorsolateral Calb1 domain appearing as late as P1 in hamster SCN, only to vanish a day after the more ventral adult Calb1 domain appears at P2. The enduring domain then enters a protracted period of maturation, achieving an adult-like appearance by ~P15 (Antle et al., 2005). Many neuropeptide populations follow similar paths and are expressed in gradually shifting, immature patterns until days or even weeks after initial detection (Delville et al., 1994; Botchkina and Morin, 1995; Herzog et al., 2000; Antle et al., 2005). Even stereotypic morphological differences in individual rat core/shell neurons do not appear until around ~P6-P10 (Moore and Bernstein, 1989). Synapse formation, which occurs concurrently with partitioning of the SCN, respects the emerging boundaries; the kinetics of synaptogenesis vary substantially among SCN subdomains, though still within the synaptogenesis window described for the SCN as a whole described by Moore and Bernstein (Laemle et al., 1991).

Against the backdrop of the long SCN maturation period in early postnatal life, non-cell-autonomous regulation of particular SCN lineages has been observed. Rat pups exposed to a pharmacological inhibitor of synthesis for the SCN input serotonin (5HT) from E13-E21 have more Vip and possibly Avp neurons, and increased cellular expression levels of these neuropeptides, in the SCN; however, this effect does not occur if the pups are instead treated with the drug early in post-natal life (Ugrumov et al., 1994; Mirochnik et al., 2005). Paradoxically, similarly increased numbers of Vip and Avp neurons and neuropeptide levels in SCN were observed in monoamine oxidase A null mice, which have constitutively elevated levels of monoamine neurotransmitters including 5HT. Increases in neuropeptide expression levels, but not cell number, could be reversed by 5HT or norepinephrine inhibitors administered post-natally (Vacher et al., 2003). One possible rationalization of these results is that embryonic inhibition of 5HT synthesis causes homeostatic up-regulation in other monoamine pathways to compensate. In another set of studies, mice selectively deficient for PSA-NCAM expressed in the dorsal SCN have three times the usual number of Vip neurons, in an expanded vlSCN (Shen et al., 1997, 1999). Thus, both PSA-NCAM and monoamine neurotransmitters are most likely regulating the differentiation of SCN neuropeptide lineages. However, another more intriguing possibility is that the overall fate of SCN neurons may remain plastic during their maturation, and can be manipulated by these extracellular cues.

Plasticity has been shown in frog SCN, where physiological light levels can dynamically induce dopaminergic marker gene expression in Npy neurons of the frog SCN (Dulcis and Spitzer, 2008). In adult rats, switching of dopamine and somatostatin expression also occurs in other parts of the AH in response to circadian light cycle changes, though such phenomena have not yet been observed in the SCN itself in mammals (Dulcis et al., 2013). However, adult mouse SCN expresses protein markers common to immature and often neurogenic cell populations, including Sox2, transportin-1, doublecortin-like, and possibly doublecortin (Geoghegan and Carter, 2008; Sato et al., 2011; Saaltink et al., 2012; Hoefflin and Carter, 2014) and SCN epigenetic state can be enduringly modified by experience (Azzi et al., 2014). Extreme plasticity of circadian behavioral entrainment is also possible in mice as late as ~P90 (Aton et al., 2004). Thus, it seems likely that SCN cell identity is at least somewhat plastic in response to circadian changes during maturation, and perhaps into adulthood.

SCN astrocyte maturation culminates in a second major jump in Gfap expression between P20-P25 in rats, shortly after the SCN neural network reaches a largely adult state (Figure 3F; Munekawa et al., 2000). Given the well-documented control of neuronal synaptic connectivity by SCN astrocytes and increased neuron-to-neuron apposition in the SCN when astrocyte maturation is disrupted by eye enucleation, a mature astrocyte network may bring relative functional stability to the SCN (Munekawa et al., 2000; Becquet et al., 2008; Girardet et al., 2010). Yet even in adulthood, it is unclear to what extent SCN development is truly fixed when presented with circadian challenge, given the possible contribution of environmentally triggered transdifferentiation and/or induced neurogenesis that have not been closely examined.

## Assembling the components: development of SCN connectivity

### Connectivity of the adult SCN

The mammalian SCN receives bilateral input of luminance information via the retinohypothalamic tract (RHT) from intrinsically photosensitive ganglion cells of the retina (ipRGCs) that express the photopigment melanopsin (Opn4) (Provencio et al., 2000; Gooley et al., 2001; Hattar et al., 2002, 2006). IpRGC input is essential for SCN light entrainment, but not clock function. *Opn4^DTA/DTA^* mice selectively lacking ipRGCs largely phenocopy the circadian effects of *Math5*^−/−^ mice that fail to develop the entire RHT due to gross retinal malformation—both animals wheel-run under light:dark (LD) conditions on an internal circadian period similar to normal mice, as though they are in constant darkness (DD) (Brzezinski et al., 2005; Guler et al., 2008). ipRGC-derived signals such as synaptically released glutamate and the neuropeptide Pacap mediate this entrainment (Chen et al., 1999; Guido et al., 1999; Hannibal et al., 2008; Kawaguchi et al., 2010; Webb et al., 2013).

The SCN is also intricately interconnected with many other regions of the brain (reviewed in Morin, 2013). One of the most prominent of these afferents comes from the thalamic intergeniculate nucleus (IGL), which sends both indirect photic information and non-photic information such as arousal state to the SCN (Pickard et al., 1987; Hastings et al., 1997; Hattar et al., 2002). The rat SCN also receives prominent inputs from the brainstem raphe nuclei, which commonly converge with IGL innervation on SCN neurons; occasionally these IGL and raphe inputs even form axo-axonic synapses with each other (Guy et al., 1987; Migliarini et al., 2013).

Other major SCN afferents include the olfactory and limbic systems, circumventricular organs, and assorted hypothalamic nuclei, among other regions (Krout et al., 2002). Interestingly, some regions like the lateral septum that project to the mammalian SCN are photosensitive in non-mammalian species (Silver et al., 1988; Li et al., 2004), suggesting that these connections may have routed photic information to our evolutionary ancestors' SCNs. The functions these formerly photic afferents serve in the mammalian circadian network are poorly understood.

SCN efferents innervate many hypothalamic nuclei, including most that reciprocally project to rat and hamster SCN. Prominent targets include the preoptic, paraventricular (PVN), subparaventricular (SPZ), retrochiasmatic, and dorsomedial (DMH) nuclei (Watts and Swanson, 1987; Watts et al., 1987; Morin et al., 1994). There are also major SCN projections to the septum and anterior periventricular thalamus, among other regions (Watts and Swanson, 1987; Watts et al., 1987; Morin et al., 1994). Efferent projections from the SCN to specific hypothalamic nuclei have been implicated in regulating circadian control of distinct subsets of behaviors. These include body temperature for the dorsal SPZ; locomotor activity levels and the sleep/wake cycle for the ventral SPZ; and locomotor activity, sleep, feeding, and stress hormone secretion for the DMH (Lu et al., 2001; Chou et al., 2003; Abrahamson and Moore, 2006).

Reflecting such differences, the afferents and efferents of different SCN subpopulations vary substantially to fulfill their divergent functions within the network. ipRGC innervation is particularly prominent for Vip neurons in the vlSCN, reflecting their key role in light entrainment, though all SCN subdomains receive at least some direct ipRGC input in mammals (Ibata et al., 1989; Tanaka et al., 1993; Muscat et al., 2003; Hattar et al., 2006; Morin et al., 2006; Juarez et al., 2013). Other major afferents, such as IGL-derived Npy and 5HT from the raphe, also preferentially innervate the vlSCN in a closely overlapping pattern, with synaptic terminals seen on Vip neurons (Kiss et al., 1984; Bosler and Beaudet, 1985; Hisano et al., 1987; Abrahamson and Moore, 2001). Conversely, septal neurons project selectively to SCN Avp neurons (Oldfield et al., 1985). Downstream, mammalian SCN target regions are generally innervated by neurons from multiple anatomical subdivisions and/or neuropeptide subtypes, though particular lineages predominate in some areas, such as the prominent Vipergic innervation of PVN (Dai et al., 1997; Abrahamson and Moore, 2001; Leak and Moore, 2001; Kriegsfeld et al., 2004; Zhang et al., 2009; Campos et al., 2014). Intra-SCN connectivity is also enormously complex and heterogeneous, and in some cases controversial (van den Pol and Gorcs, 1986; Daikoku et al., 1992; Ibata et al., 1993; Romijn et al., 1997; Jacomy et al., 1999; Castel and Morris, 2000; Abrahamson and Moore, 2001; LeSauter et al., 2002). In short, correct control of developing SCN connections is likely every bit as important as expression of the proper neuropeptides in establishing adult SCN heterogeneity and function.

### Development of SCN afferents

By far the most heavily studied SCN afferent is the ipRGC input. ipRGCs are born in a much wider window than SCN neurons, extending from ~E11-E18 in mouse (McNeill et al., 2011). Opn4 is initially expressed at about 75% of gestation, or ~E15 in mice and ~E18 in rats, roughly concurrent with the end of SCN neurogenesis (Fahrenkrug et al., 2004; McNeill et al., 2011). IpRGC axons are present at the mouse optic chiasm by E17, but for reasons that are unclear they stall here for several days before innervating the SCN, lagging RGC innervation of brain regions involved in image-forming vision (McNeill et al., 2011).

**Figure 4:**
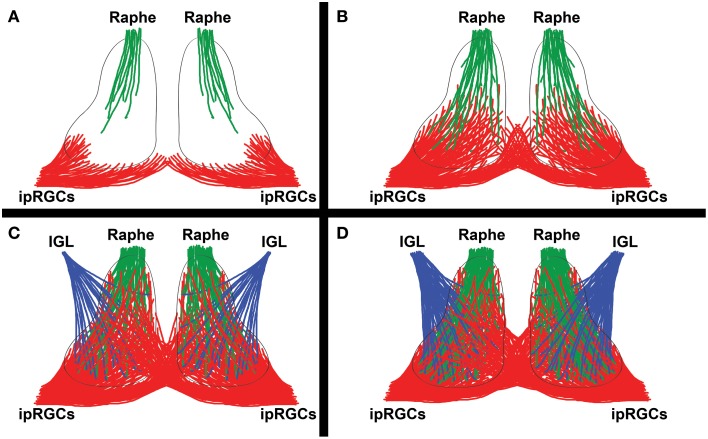
**Innervation by major SCN afferents**. A simplified diagram showing the development of major SCN afferents from the intrinsically photosensitive retinal ganglion cells (ipRGCs) of the retina, raphe nuclei, and intergeniculate leaflet (IGL). **(A)** ipRGCs first begin to innervate the vlSCN ipsilaterally at birth (mouse/rat P0-P1) or shortly thereafter (hamster P3). The raphe nuclei first innervate the SCN simultaneously (mouse P0, hamster P3). **(B)** A few days later, ipRGC innervation of the SCN becomes noticeably denser and broader, with the first contralateral projections appearing in the ventromedial SCN (mouse/rat P4), as raphe innervation also builds. **(C)** By roughly a week into postnatal SCN development, raphe innervation has become fairly dense (mouse P5, hamster P6), ipRGC contralateral and ipsilateral ipRGC innervation domains are commingled (mouse P7), and the earliest Npy signal from the IGL is detectable (hamster P7, rat P10). Many of the IGL and raphe projections converge, a trend that persists through adulthood. **(D)** In the following few weeks, more-or-less adult SCN innervation by the raphe (mouse P10), ipRGC (mouse P14), and IGL (rat P20) projections become apparent.

There are substantial species differences in the rate of ipRGC innervation of SCN across mammals. The vlSCN is only gradually innervated around birth in mice and rats, but because of their Opn4 expression, ipRGCs are light responsive and able to activate a subset of vlSCN neurons that they sparsely innervate from P0-P1 onwards (Figure 4A; Speh and Moore, 1993; Hannibal and Fahrenkrug, 2004; Sekaran et al., 2005). Initially, the ipRGCs only innervate the vlSCN ipsilaterally in mouse, but as the projection matures the entire SCN is innervated, with contralateral projections appearing by P4 and becoming commingled with the ipsilateral projections by P7 (Figures 4B,C; McNeill et al., 2011). In rats, the diversity of individual ipRGC-SCN synapses increases dramatically during this time (Lenn et al., 1977). In hamsters this process is slightly delayed but fundamentally similar to the pattern in mice and rats, with initial ipRGC innervation and activation of vlSCN occurring at ~P3 (Speh and Moore, 1993; Kaufman and Menaker, 1994; Duffield et al., 1999). Remarkably, all of these events occur before eye opening in all three species.

By ~P10 (mouse/rat) or ~P15 (hamster), the ipRGC projection to SCN is mostly mature, with largely adult-like projection density, morphology of individual axon terminals, and light responses (Figure 4C; Moore and Bernstein, 1989; Speh and Moore, 1993; Kaufman and Menaker, 1994; Hannibal and Fahrenkrug, 2004; Sekaran et al., 2005; McNeill et al., 2011), though some maturation continues at least through adolescence (Yellon et al., 1993). During or shortly after the mostly mature P10-P15 range in rodents, rod and cone photoreceptors become mature and the eyes open, rendering Opn4 largely redundant for SCN photoentrainment (Panda et al., 2002; Hannibal and Fahrenkrug, 2004; Sekaran et al., 2005).

The maturation of ipRGC inputs to the SCN in early post-natal life has important implications for SCN development. Multiple NMDA receptor subunits are transiently expressed between birth and eye opening in rat and hamster SCN (Bendova et al., 2009; Duffield et al., 2012), suggesting a period during which SCN development is sensitive to ipRGC-derived glutamate. IpRGC innervation is necessary for certain events in mouse SCN differentiation, such as Calb1 downregulation in dmSCN and Calb2 upregulation in vlSCN during early postnatal life (Ikeda and Allen, 2003), as well as SCN astrocyte maturation (Munekawa et al., 2000). Manipulations of the light cycle during this time also influence SCN development and adult function—rearing in DD decreases adult SCN neuron and glia numbers, while rearing in constant light (LL) decreases adult SCN glia numbers (Cambras et al., 2005). There have also been reports of differences in adult SCN Vip and Avp immunostaining intensity and circadian behavior in rats and mice exposed to postnatal light manipulations; however, the findings have varied substantially, possibly as a result of species-specific differences or variations in experimental protocol (Smith and Canal, 2009; Cambras et al., 2015).

Thus, exceptions to this pattern of gradual postnatal ipRGC innervation of SCN are both developmentally and evolutionarily interesting. For example, in stark contrast to more commonly studied rodents, rabbits are born with an over-abundant ipRGC projection to SCN that is pruned down to adult levels by P19 (Juarez et al., 2013). Though the mechanisms behind this difference are presently unclear, the ultimate cause may relate to the maternal behavior of rabbit dams, which visit the burrow to suckle pups for a much shorter time each day than rodents such as mice and hamsters (Zarrow et al., 1965). Although the SCN-independent food-entrained circadian system is likely most important for anticipating the dam's visit (Caba et al., 2008), the ipRGC projection may have nonetheless evolved to become particularly exuberant in the early postnatal stage, to aid in correctly timing the all-important daily feeding. The impact this very different time-course of ipRGC development may have on development of the rabbit SCN is an intriguing proposition.

After ipRGCs, the most extensively studied afferent projection from a developmental perspective is the ascending raphe projection, which is one of the last raphe efferent projections to form in hamsters. Its inputs to the SCN initially arrive in the SCN at times similar to the ipRGCs, by ~P0 in mouse and ~P3 in hamster, and their maturation process seems to take a similar amount of time (Figures 4B–D; Botchkina and Morin, 1993; Migliarini et al., 2013). Co-transplantation studies suggest that raphe innervation of the rat SCN may require IGL innervation of the SCN to form; if the same is true in normal development, it would explain the frequent convergence of these afferents in the adult SCN (Guy et al., 1987; Ueda et al., 1993, 1995). The SCN is also one of the very few nuclei that requires serotoninergic signaling by raphe neurons in order to receive raphe innervation (Migliarini et al., 2013). As mentioned previously, the raphe projection is also involved in regulating the number of Vip and (possibly) Avp cells in the SCN, a relationship that is likely dependent on the formation of direct synaptic contacts between 5HT terminals and Vip neurons in the SCN (Kiss et al., 1984; Bosler and Beaudet, 1985; Hisano et al., 1987; Mirochnik et al., 2005).

A bit is also known about the development of IGL projections to the SCN. Npy is detectable in the SCN by ~P7 in hamster and ~P10 in rat, suggesting that IGL projections are present by this time (Figure 4C; Romero and Silver, 1990; Takatsuji et al., 1995). This is markedly delayed relative to the early presence of the ipRGC and raphe projections; however, the probable IGL role in raphe innervation of the SCN hints that the IGL projection to SCN may be present much earlier in development than Npy is detectable, a possibility that has not been directly explored (Ueda et al., 1995). In any case, by P20 the IGL projection to SCN is largely mature in rats (Figure 4D; Takatsuji et al., 1995). To our knowledge, the underlying mechanisms and developmental role of this projection have not been studied, beyond the aforementioned transplantation study by Ueda et al. Very little is known about the development of other SCN afferents, aside from the three discussed above.

### Development of SCN efferents and paracrine signaling

Virtually nothing is known about the development of SCN efferents. In hamster SCN, one DiI tracing study suggests that efferent growth begins at ~P1, with rapid, largely simultaneous growth toward known SCN target regions (Muller and Torrealba, 1998). This is the only such study we were able to locate.

The surprising lack of data on this topic may stem from the perceived importance of paracrine signals for adult SCN output. The foundation of this long-controversial point is that fetal SCN grafts wrapped in a mesh to block efferent outgrowth are sufficient to rescue circadian activity rhythms in SCN-lesioned hamsters (Silver et al., 1996). However, while both the rat SCN nucleus and its efferents are essential for sleep-wake rhythmicity at P8, SCN efferents but not the SCN nucleus are partially dispensable in younger P1 animals (Gall et al., 2012). Meanwhile, neonatal mouse *Cry1^−/−^;Cry2*^−/−^ SCN is surprisingly able to sustain synchronized Per1 and Per2 luciferase (Luc) rhythms for a few days *in slice*; this ability is lost by ~P7, after which the mutant SCN is arrhythmic as soon as it is explanted (Maywood et al., 2011; Ono et al., 2013). While a neonatal wild-type or *Cry1^−/−^;Cry2*^−/−^ SCN can entrain a co-cultured arrhythmic adult *Cry1^−/−^;Cry2^−/−^* SCN in a paracrine fashion, an adult wild-type SCN cannot (Ono et al., 2013). And tellingly, in hamster SCN implantation studies, efficacy is highly correlated with donor age, with ~P7 or older grafts failing to efficiently entrain rhythms (Romero et al., 1993).

In sum, it seems likely that SCN-derived paracrine signaling may indeed be sufficient for behavioral entrainment for a transient period during early postnatal development. However, at least in slice preparations, the SCN neuropeptides that are most highly expressed in adult animals–such as Vip, Grp, and Avp–account for much of the nucleus' paracrine entraining capacity (Maywood et al., 2011). One possible hypothesis rationalizing this seeming paradox is that a modifier of adult neuropeptide signaling is downregulated in early postnatal life, causing a loss of SCN paracrine signaling competence. Possible candidates include regulator(s) of paracrine release or proteolytic enzyme(s) that alter neuropeptide precursors post-transcriptionally. In any case, both the study of SCN paracrine signaling and efferent outgrowth in a developmental context are clearly fruitful areas for future research.

## Winding the watch: ontogeny of SCN clockworks

### Clock diversity in adult SCN

Mirroring the heterogeneity in SCN gene expression and connectivity, the SCN clockworks are quite complex, with oscillatory clock gene expression varying substantially across different subdomains (reviewed in Honma et al., 2012). For example, a number of Per1Luc and Per2Luc slice studies in both rats and mice show that intrinsic dmSCN rhythms are phase-advanced relative to vlSCN rhythms (Yamaguchi et al., 2003; Foley et al., 2011; Fukuda et al., 2011), likely due in part to shorter intrinsic periods in dmSCN (Noguchi et al., 2004). Conversely, phase-shifts induced by light begin in the vlSCN, before spreading to the dmSCN (Nagano et al., 2003). The phase of subdomain clock gene rhythms also varies across the anteroposterior axis of the mammalian SCN in a manner that, though it depends somewhat upon light cycle, under most conditions is phase-advanced in posterior SCN relative to central and anterior subdomains (Hazlerigg et al., 2005; Sosniyenko et al., 2009; Evans et al., 2011). Variation along this axis phase-locks to the onset and offset of behavioral rhythms, implying behavioral relevance (Inagaki et al., 2007).

Divergent patterns are also noticeable in readouts of SCN activity. There are dramatic differences in the phase of single-unit mouse SCN recordings that strongly correlate with regional variation in Per1GFP rhythms seen across the SCN (Quintero et al., 2003; Schaap et al., 2003). SCN calcium rhythms show a similar pattern, at least insofar as dmSCN is phase advanced relative to vlSCN (Enoki et al., 2012; Brancaccio et al., 2013). Furthermore, cultured rat SCN shows that the circadian rhythm of Avp release is phase-advanced relative to Vip, when the latter is rhythmic (Nakamura et al., 2001). Coding of circadian parameters by SCN neuron activity also varies across compartments; for instance, while population differences in the phase of single-units can be compressed or decompressed by light cycle to encode period, this appears to occur preferentially in the vlSCN, while dmSCN relies more on changes in the period of individual oscillators to encode period (VanderLeest et al., 2007; Brown and Piggins, 2009).

Beyond these subdomains, there is also considerable cell-to-cell variation in the period and amplitude of individual oscillators, and even whether a cell is rhythmic or not. This is true for both clock gene expression (Herzog et al., 2004; Webb et al., 2009) and cellular activity (Nakamura et al., 2001; Herzog et al., 2004; Honma et al., 2004; Webb et al., 2009).

Both cell fate and connectivity play important roles in establishing clock diversity. The period of Bmal1 rhythms varies subdomain-specifically and intrinsically encodes period in mouse SCN neurons, and temporal organization of SCN synchrony seems to arise predominantly from cell-autonomous factors (Myung et al., 2012; Pauls et al., 2014). On the other hand, spatial organization of SCN synchrony is primarily signaling-dependent, likely due to features such as dye coupling of cells within but not between vlSCN and dmSCN, the influence of Vipr2 signaling on Avp neuron period, and the Gαi-protein inhibitor Rgs16's role in driving circadian oscillations of cAMP production in the SCN (Colwell, 2000; Noguchi and Watanabe, 2008; Doi et al., 2011; Pauls et al., 2014). However, these aspects of cell identity seem less important for whether the SCN as a whole is rhythmic or not, despite effects on the parameters of cells that are rhythmic (Webb et al., 2009). The development of this complex clockwork will be the focus of the final section.

### Clock ontogeny in developing SCN

**Figure 5:**
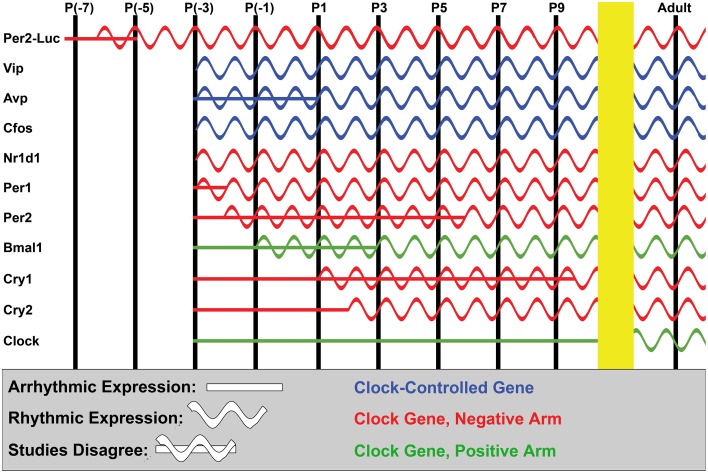
**Ontogeny of the SCN clockworks**. A simplified diagram showing the approximate age at which expression and cycling of various cellular clock and clock-controlled genes are known to begin in the SCN. Data from all mouse and rat studies we were able to locate is included in the figure; our P(-x) terminology is used for prenatal time-points to roughly account for the difference in average gestation time in mouse (P0 at ~20 days) and rat (P0 at ~22 days). *In vivo* studies were surprisingly consistent despite differences in organism and detection methodology among studies, arriving at estimated ages of rhythm ontogeny within a few day window in most cases. Notably late outliers include Per2 (Shimomura et al., 2001) and Cry1 (Ansari et al., 2009). New studies observing Per2-Luc luminescence *in slice* have arrived at earlier estimates of rhythm induction than *in vivo* studies of Per2; however, the reason for this is unclear (see text). Finally, note that only induction of expression and rhythmic expression are accurately recapitulated in this figure; relative phase and amplitude of the peaks is incidental.

It has recently become clear that Per2Luc signal in mouse SCN slices is rhythmic in culture as young as either E13.5 or E15, as found by two different groups (Wreschnig et al., 2014; Landgraf et al., 2015). Regardless of which age of onset is correct, both studies detect Per2 rhythmicity much younger than the earliest detectable SCN clock gene rhythms in previous *in vivo* studies (Figure 5). One possible explanation is that individual mouse SCN neurons begin to function as isolated oscillators sometime between E13.5-15, which are prematurely aligned to produce rhythms by the synchronizing cue of the culturing process. Supporting this interpretation, early SCN rhythms are unique among multiple tissues studied by Landgraf et al. in strongly phase-locking with culturing time (2014a), consistent with earlier studies showing an elevated dependence of SCN phase on this parameter when cultured from early post-natal mice (Nishide et al., 2008). Alternatively, it is possible that Per2Luc signal amplification simply allows more sensitive detection of rhythmicity, as earlier studies of clock ontogeny *in vivo* relied upon histological techniques that are less sensitive to very small changes in expression levels. However, a recent *in vivo* study using more sensitive quantitative real-time PCR methods also found later onsets of rhythmicity more in keeping with the older histological data (Houdek and Sumová, 2014), making it less likely that differences in detection limits are the sole cause of this discrepancy. The data from these Per2Luc studies remains interesting and important in either case, but clarifying their interpretation remains an important future challenge.

*In vivo*, many clock and clock-controlled genes are robustly expressed in the mammalian SCN by ~E13 in hamster, ~E17 in mice, and ~E19 in rat (Figure 5; Shearman et al., 1997; Sladek et al., 2004; Li and Davis, 2005). The SCN clockworks become synchronously rhythmic in a staggered fashion shortly thereafter. Non-core-clock and clock-controlled SCN-enriched gene expression, such as *Nr1d1, Vip, Avp*, and *c-fos* mRNA, generally become rhythmic first, by ~E19 in rat and late embryonically in mouse (Figure 5; Huang et al., 2010; Houdek and Sumová, 2014). This corresponds to the onset of rat SCN metabolic rhythms at ~E19, implying that the core clockworks are dispensable for this functional rhythm (Reppert and Schwartz, 1984). Interestingly, reports largely concur that core clock genes are not yet synchronously rhythmic in the developing SCN at this age *in vivo* (or at least that the rhythms are of quite low amplitude), suggesting that the maternal clock initially drives synchrony of developing pups' SCN clock-controlled gene expression *in vivo*, perhaps via modulation of *Nr1d1* expression (Houdek and Sumová, 2014).

Genes in the negative arm of the core clock become rhythmic next. In some studies, *Per1* and *Per2* are among the earliest to begin cycling, at ~E17 in mouse and ~E20 in rat (Figure 5; Shearman et al., 1997; Shimomura et al., 2001; Ohta et al., 2002; Kovacikova et al., 2006), while in other studies in mouse and hamster, *Cry1* is among the earliest rhythmic clock components (Li and Davis, 2005; Huang et al., 2010). Genes in the positive arm become rhythmic later; Clock is particularly notable in this regard, as it does not begin cycling until sometime after P10 in both mouse and rat (Figure 5; Sladek et al., 2004; Kovacikova et al., 2006; Ansari et al., 2009). Most clock genes are rhythmic by ~90% of gestation across mammals and all become rhythmic within the first several post-natal days, though details vary somewhat among reports (Figure 5; Sladek et al., 2004; Li and Davis, 2005; Kovacikova et al., 2006; Torres-Farfan et al., 2006; Huang et al., 2010; Houdek and Sumová, 2014). SCN neural activity rhythms also appear within this window, at ~E22 in rat SCN (Shibata and Moore, 1987). Combined with the strong correlation between local clock gene expression and adult SCN neural activity (Quintero et al., 2003), it seems that the core clock is required for induction of rhythmic SCN neural activity.

Following their initial emergence, SCN rhythms mature in several ways. Overall expression level of rhythmic genes is divergent in late embryonic mouse SCN, with genes that become rhythmic early increasing and genes that become rhythmic later decreasing in expression (Houdek and Sumová, 2014). Rhythmicity of most core clock and clock-controlled genes in the SCN is initially much weaker than in adults and strengthens throughout late embryonic and early post-natal life, reaching adult or near-adult amplitudes at ~P10 for most genes, excepting Clock (Sladek et al., 2004; Li and Davis, 2005; Kovacikova et al., 2006; Sumová et al., 2006; Ansari et al., 2009; Huang et al., 2010; Houdek and Sumová, 2014; Wreschnig et al., 2014). Rat SCN firing rhythms reach adult amplitudes shortly thereafter, at ~P14 (Shibata and Moore, 1987). The notable exception to increasing rhythmicity with age is Vip, which becomes steadily less rhythmic during post-natal development even as its expression level rises, reaching its diminished adult amplitude by P20 in rat SCN (Ban et al., 1997). Peak and trough phase also changes dramatically for some rhythmic SCN genes during this time, including *Cry1, Nr1d1, Avp*, and *Vip* (Ban et al., 1997; Huang et al., 2010; Houdek and Sumová, 2014).

Despite the growing body of research on clock diversity in the adult SCN, to our knowledge there are currently no studies examining the ontogeny of subdomain differences in phase and period of SCN rhythms. However, marked changes in expression patterns of some clock genes within the SCN, such as *Rorα*, between embryonic development and adulthood suggest substantial shifts in the spatiotemporal clock arrangement (Shimogori et al., 2010; Hatori et al., 2014). These whole-SCN changes during early post-natal life may indicate the existence of fruitful developmental windows to study the emergence of subdomain-specific clocks (Ban et al., 1997; Huang et al., 2010; Houdek and Sumová, 2014).

There is, however, evidence for subdomain-specific clock effects on other aspects of SCN development. For example, there are conflicting reports about SCN cell fate changes in *Clock*^−/−^ mice. In both studies, Avp neuron number was reduced prior to P6; one of the groups also reported a failure of Vip neurons to increase in number and properly localize after P6, though Vip neuron development before that age seemed normal (Silver et al., 1999; Herzog et al., 2000). There is also a selective increase in Calb1 neurons in *Tau* mutant hamsters (LeSauter et al., 1999). Thus, the developing clockworks seem to influence SCN cell fate during early post-natal life, and possibly earlier.

### Entrainment of developing SCN clocks

As the SCN clock begins ticking, it is faced with a problem: how to align itself with the solar cycle in the absence of light input in the womb. The mother entrains emerging SCN rhythms, but this requires an intact circadian system. SCN lesion of dams at gestational day 7 disrupts within-litter synchrony of SCN metabolic rhythms in E21 rats, melatonin synthesis rhythms in P10 rats, and rest/activity rhythms in P10 hamsters (Reppert and Schwartz, 1986; Davis and Gorski, 1988). Similarly, rhythmic *Per1*^±^;*Per2*^±^ or *Cry1*^±^;*Per2*^±^ mouse pups born to homozygous mutant dams and wild-type studs show reduced within-litter synchrony of activity rhythms (Jud and Albrecht, 2006).

Interestingly, disrupting maternal rhythms shortly before birth by ablating the SCN of pregnant hamster dams at gestational day 14 has a less severe effect on within-litter synchrony of pups than SCN ablation at day 7 (Davis and Gorski, 1988). Similarly, phase shifting a pregnant rat dam at gestational day 18, but not day 20, results in phase shifts in c-fos and AVP rhythms in her developing pups' SCNs (El-Hennamy et al., 2008). Postnatally, fostering blinded rat pups with a phase-reversed dam, or administering a 6-h phase shift, have only very small effects on pup SCN clock gene phase (Ohta et al., 2002), though maternal absence during defined intervals is a much more robust entraining cue (Shimoda et al., 1986; Viswanathan, 1999; Ohta et al., 2003). Thus, excepting conditions of extreme stress such as dam absence, maternal control of synchronization and phase setting of her pups' SCN rhythms rapidly decreases in later embryonic life, as the pup's own rhythms synchronize and begin to oscillate independently of the dam's cues.

But what are these cues? One perennial but controversial candidate is melatonin secreted by the pineal gland. In rat pups born to LL-housed dams, failure of SCN c-fos and Avp rhythms to form by P1 is rescued by E16-E21 melatonin injections (Houdek et al., 2014). Though vehicle injections have a similar effect, melatonin but not vehicle remains able to rescue this defect in pups born to pinealectomized, LL-housed dams (Houdek et al., 2014). Dam pinealectomy at gestational day 7 is also sufficient to disrupt within-litter synchrony of drinking rhythms in rat pups (Bellavia et al., 2006). Melatonin is also present in the milk of mammals, including humans, and exogenous melatonin is sufficient to entrain rat and hamster rhythms both embryonically and in early postnatal life (Davis and Mannion, 1988; Illnerova et al., 1993; Grosse et al., 1996; Viswanathan and Davis, 1997).

On the other hand, propanol-mediated blockade of melatonin secretion of suckling rat dams has no effect on within-litter synchrony of pup melatonin secretion rhythms, though as discussed previously, this study's intervention lagged behind the most important developmental window for maternal entrainment (Rowe and Kennaway, 2002). More tellingly, pineal melatonin secretion has been lost in most laboratory mouse strains, likely due to artificial selective pressures against seasonally regulated reproduction, with no clear effect on the development of SCN rhythmicity (Goto et al., 1989). Given this important counterpoint, while melatonin is very likely one of the maternal entraining cues in mammals, it seems to be largely dispensable, likely due to redundancy with other factors.

Dopamine is a particularly attractive candidate to play a complementary role to melatonin at this stage, given their powerful but diametrically opposed effects on both phase and the cAMP signaling system involved in adult SCN entrainment (Viswanathan and Davis, 1997; Vanecek and Watanabe, 1998; Schurov et al., 2002; An et al., 2011; Atkinson et al., 2011). Dopamine agonists are sufficient to entrain late-embryonic hamsters, and are able to induce c-fos in embryonic rat and hamster SCN with rapid, transient kinetics similar to that induced by light later in life (Weaver et al., 1992, 1995; Viswanathan et al., 1994; Viswanathan and Davis, 1997; Duffield et al., 1999). D1 dopamine receptor (D1r) is transiently expressed in rat SCN, and while *D1r* is not developmentally regulated in hamster, SCN innervation by dopaminergic afferents is (Weaver et al., 1992; Duffield et al., 1999). Accordingly, rat SCN stops responding to dopamine stimulation at ~P4, as light control of the SCN increases; however, eye enucleation fails to rescue loss of dopamine sensitivity, so different mechanisms control light sensitization and dopamine desensitization in the developing SCN (Weaver and Reppert, 1995).

This handoff between signals controlling SCN entrainment is gradual. As mentioned previously, acute SCN light activation, as measured by c-fos expression, begins between P0 and P1 and reaches adult levels by P10 in mouse and rat, and begins by P4 and matures by P15 in hamster (Kaufman and Menaker, 1994; Leard et al., 1994; Sekaran et al., 2005; Mateju et al., 2009). Acute light induction is observed by P1 for *Per1* and P3 for *Per2* in rat SCN (Mateju et al., 2009). However, phase-dependent gating of all three genes' responses to light comes significantly later: *Per1* induction is gated by P3, *Per2* by P5, and *c-fos* induction is not gated until P10 (Bendova et al., 2004; Mateju et al., 2009). The substantial delay of phase-dependent gating of c-fos induction, relative to light-dependent inducibility, supports the importance of both a fully functional SCN clockwork and neurotransmitter network for controlling SCN activity, as this coincides with the maturation of most clock gene rhythms, arrival of most afferents, and adult-like expression of many cell-specific markers within the SCN.

SCN entrainment by light also developmentally lags behind the ability of light to induce acute responses. Under 8:16 and 16:8 LD cycles, changes are not detectable in rat SCN *Per1/2* rhythms until P10 and *Cry1* rhythms until P20, while *Bmal1* rhythms are still unaffected even at P20 (Kovacikova et al., 2005; Sumová et al., 2006). Similarly, the SCN shell c-fos rhythm fails to respond in an adult pattern to a 16:8 light cycle as late as P10 (Bendova et al., 2004). This starkly contrasts with the marked plasticity of behavior in adolescent and young adult mice later in development, once light entrainment is fully on-line (Aton et al., 2004). Thus, the late developmental onset of full SCN light entrainment may be necessary to prevent maladaptive alterations of the SCN's homeostatic range during the period when its cell fate, connectivity, and clockworks are still extremely plastic, prior to the full maturation of the SCN astrocyte network and/or other stabilizing factors (Munekawa et al., 2000).

## Conclusion

In service to the SCN's role as the master light-entrained oscillator, SCN neurons have numerous signals, connections, and clock features that work together, with more advanced functions of the central clock requiring progressively more communication within this heterogeneous network. But for this enormously complex master clock to begin ticking, the gears must be appropriately forged, interlocked, and wound during the development of the organism. Importantly, none of these steps can be accomplished without allowance for the others. Misshapen gears will not interlock. Poorly fitted gears will destroy each other. And a perfectly assembled but unwound clock is useless.

The dramatically differing traits of vlSCNmain and vlSCNmed neurons in the adult rat SCN serve as a perfect exemplar of this point. While vlSCNmain cells are densely innervated by ipRGCs, show Per1 induction by light, and rhythmically express Vip that is often co-expressed with Grp, vlSCNmed cells have little ipRGC innervation, show no Per1 induction by light, and express Vip in a pattern that is neither rhythmic nor co-expressed with Grp (Kawamoto et al., 2003). All of these differences in gene expression, connectivity, and clockworks are consistent within the respective populations and, given this consistency, are presumably important for their respective functions. But to ensure that the appropriate traits co-localize, the development of these various traits must be coordinated.

Thus, although we have separated our discussions of how cell fate, connectivity, and clock ontogeny emerge during SCN development, we also hope to have conveyed the profound integration of these processes. Not coincidentally, this mirrors the current situation in the field. Historically, the study of these topics was limited by a poor understanding of hypothalamic development in general, which prevented genetic tools being pioneered in the cortex and other brain regions from being applied in the SCN. But now, recent large-scale studies mapping hypothalamic gene expression (e.g., Lein et al., 2007; Shimogori et al., 2010; VanDunk et al., 2011) and the burgeoning availability of Cre/lox and other alleles for intersectional genetic approaches in mice have dramatically improved our ability to attack these questions. This directly prompted the ongoing burst of progress in SCN development, particularly the roles of various transcription factors in the process that are discussed in this review and elsewhere (Landgraf et al., 2014).

The interrogation of Lhx1's role in the SCN is a case in point. Constitutive mutants for Lhx1 fail to form anterior head structures entirely (Shawlot and Behringer, 1995), precluding study of the SCN in these animals. It was only by targeting a loxP-flanked Lhx1 allele with Cre drivers whose overlaps with Lhx1 expression are constrained to the developing vAH and retina (Six3-Cre) or SCN and cerebellum (Rorα-Cre) that studies of this gene's role in several aspects of SCN development and function became possible (Zhao et al., 2007; Shimogori et al., 2010; Bedont et al., 2014; Hatori et al., 2014; Allen Brain Atlas).

As such approaches are adopted more broadly in hypothalamic developmental studies, the benefits for understanding the basic biology of the adult SCN will be three-fold. First, developmental mutants that survive to adulthood often present unique and interesting phenotypes difficult to obtain in other ways, such as the loss of multiple neuropeptides in the *Lhx1*-deficient SCN. Second, by characterizing the spatial and temporal expression of ever more SCN-enriched genes, developmental studies will also steadily improve our ability to intersectionally target genes of interest in the adult SCN. And finally, the study of SCN development in multiple species has given intriguing insights into the evolution of the region, providing tantalizing clues about both the “hows” and “whys” of SCN function.

That said, most work on mammalian SCN development has been conducted in rodent species (predominantly mice, rats, and hamsters), raising some questions about the extent to which this body of work extends to mammals at large, including primates. What is known suggests that the broad strokes of findings in rodents are generalizable within the mammalian clade. The basic structure of the adult SCN and its suite of neuropeptides is largely preserved with mostly modest variations in mammals as diverse as mice, cats, cows, pigs, rhesus macaques, and humans (Sofroniew and Weindl, 1980; Stopa et al., 1984; Cassone et al., 1988; Dai et al., 1997; Abrahamson and Moore, 2001), with the notable exception of the re-emergence of local SCN Npy expression in humans (Moore, 1989). Furthermore, the evidence we discussed earlier suggesting that Lhx1 controls SCN neuropeptide fate in both amphibians and mammals, despite substantial changes in adult SCN neuropeptide composition between these clades, implies that the genetic regulatory networks controlling SCN formation are even better conserved across species than the SCN's adult form (Tuinhof et al., 1994a,b; Abrahamson and Moore, 2001; Brohl et al., 2008; Dulcis and Spitzer, 2008; Bedont et al., 2014).

And perhaps most importantly, the phenomenology of many SCN developmental events is similar in rodents and primates. Sequential induction of SCN neuropeptide protein expression remains the rule in humans, and according to most studies the gestation-adjusted age of initial detection in humans is similar to rodents at ~80% of gestation for Vip and around birth for Avp (Swaab et al., 1990, 1994), though others claim to observe somewhat earlier but similarly sequenced induction (Xu et al., 2003). And much like in rodents, SCN clock ontogeny starts at ~90% of gestation in capuchin monkeys, with Bmal1 and Per2 cycling beginning at P142 (Torres-Farfan et al., 2006). These similarities with the rodent developmental timeline hold despite SCN neurogenesis occurring between E27-E48 (~16–29% of gestation) in rhesus macaques, with the densest production from E30-43, dramatically earlier on a gestationally adjusted basis than in rodents (van Eerdenburg and Rakic, 1994). In sum, the available data suggests that primate SCN development is drawn out over a significantly longer time than, but is mechanistically similar to, rodent SCN development, ultimately producing a functionally similar SCN at correspondent developmental stages. That said, with genetic tools in marmosets coming online in recent years, a direct examination of whether primate SCN developmental mechanisms are fundamentally similar to rodents by intersectional loss-of-function studies is a promising proposition. This is due not only to the topic's profound implications for the basic biology of SCN development and evolution, but also for human health.

Circadian disruption over the course of development in humans is very common. For instance, during adolescence the prevalence of formal delayed sleep phase disorder (DSPD) is estimated at ~7–16% (reviewed in Bartlett et al., 2013), and many teenagers that do not meet this formal diagnosis nonetheless tend toward a “night owl” chronotype. Coupled with social expectations out of phase with biological reality, this circadian change likely contributes to the incidence of psychiatric illness in adolescents, as DSPD and sometimes even the night owl chronotype alone have been linked to depression, obsessive-compulsive disorder, and other mental ailments (Lewy, 2009; Reid et al., 2012; Schubert and Coles, 2013). Early attempts at interventions in a military basic training environment have suggested that simply adapting adolescents' schedules to compensate for their altered chronotype improves sleep quality and learning, while ameliorating mood disturbance (Miller et al., 2012). A better understanding of SCN development may give insight into new approaches and tools for manipulating the circadian system for psychiatric effect during adolescence and at various other developmental stages.

Later in life, low amplitude and late phase of sleep/wake rhythms are predictive of problems such as dementia (Tranah et al., 2011) and progressive degradation of sleep/wake rhythms is apparent in both Alzheimer's and Huntington's disease (Harper et al., 2004; Morton et al., 2005). Age-related circadian dysfunction likely stems at least partially from disruptions in SCN clock output; while core clock gene rhythms remain robust in the SCN throughout the lifespan in rodent models, output-related clock gene expression, electrical activity, and neuropeptide rhythms all decline with age (Roozendaal et al., 1987; Kawakami et al., 1997; Nakamura et al., 2011; Farajnia et al., 2012; Bonaconsa et al., 2014). Similar deficiencies have been noted in the aged human SCN, and many of these deficits are exacerbated in dementia patients, supporting the view that organic decline of the SCN contributes to the late-life sleep/wake fragmentation linked to neurodegeneration (reviewed in Hofman and Swaab, 2006). Understanding how various aspects of SCN output come online during development may give insight into how to restore them later in life, to combat this decline.

Finally, a thorough understanding of SCN cell fate determination is also a prerequisite to differentiate induced pluripotent stem cells (iPSCs) into SCN neurons. Aside from neurodegenerative and psychiatric disease, circadian dysfunction is implicated in a host of metabolic and cardiovascular disorders in humans, as well as cancer and obesity (reviewed in Campos Costa et al., 2013; Froy, 2013; Videnovic et al., 2014; Virag and Lust, 2014; Masri et al., 2015). The ability to differentiate a human SCN from iPSCs will allow the modeling of such patients' central clock in culture, aiding in the identification of abnormalities in SCN function that may contribute to their pathology. iPSC-derived SCN transplants also represent a potential future cure for explicit disorders of the core circadian clock, as such transplants impart their period to the host's circadian behavior in animal studies (Ralph et al., 1990). One such condition is familial advanced sleep phase disorder, a genetic ailment in which clock gene mutations disrupt the patient's internal period (reviewed in Nesbitt and Dijk, 2014). Such an intervention would free these patients from the constant need to rigidly structure their lives, in an effort to shoehorn their misaligned rhythms into following the solar cycle.

In conclusion, we now stand at the edge of a promising new path forward to study SCN development, illuminated by new technologies and following paths laid down in decades of invaluable descriptive studies. As our understanding of the region's developmental program accumulates in the years to come, so too will our understanding of how and why various individual traits of SCN neurons are bound together in the adult network to produce the central circadian clock. And in the process, we stand to gain unique insight not only into how our internal watch is made, but also in how to repair it as time and the environment takes its toll.

### Conflict of interest statement

The authors declare that the research was conducted in the absence of any commercial or financial relationships that could be construed as a potential conflict of interest.

## Footnotes

^1^ Allen Brain Atlas (http://www.brain-map.org/). Accessed Sept 1-Dec 31, 2014.

## Abbreviations

5HT: serotonin (raphe neurotransmitter)
Avp: arginine vasopressin (SCN neuropeptide)
Avpr1a/1b: Avp receptors 1a/1b
Bmal1 (Arntl): basic helix-loop-helix-PAS domain transcription factor (clock gene, core positive arm)
Calb1: calbindin, calcium binding protein
Calb2: calretinin, calcium binding protein
c-fos: proto-oncogene (induction of gene expression is indicative of neuron activation)
Clock: basic helix-loop-helix-PAS domain transcription factor (clock gene, core positive arm)
Creb3l1: cAMP responsive element binding protein 3-like 1 transcription factor
Cry1/2: cryptochrome 1/2 (clock gene, core negative arm)
D1r: dopamine receptor 1
DD: constant darkness
dlAH: dorsolateral anterior hypothalamus (developmental compartment that gives rise to neuroendocrine hypothalamus)
DMH: dorsomedial hypothalamus
dmSCN: dorsomedial shell subdomain of the SCN, classically defined by Avp expression
E(x): embryonic day x of gestation
Foxd1: forkhead box transcription factor D1
Fzd5: frizzled 5, Wnt receptor
Gfap: glial fibrillary acid protein (astrocyte marker)
Grp: gastrin releasing peptide (SCN neuropeptide)
IGL: intergeniculate leaflet of the thalamus (major SCN afferent)
ipRGC: intrinsically photosensitive retinal ganglion cells (major SCN afferent)
LD: light:dark entrainment cycle
Lhx1/2/8: LIM homeobox transcription factor 1/2/8
Math5 (Atoh7): basic helix-loop-helix transcription factor
Nkx2.2: NK2 homeobox transcription factor 2.2
Npy: neuropeptide Y (IGL neuropeptide)
Nr1d1 (Rev-erbα): nuclear receptor 1 d1 (clock gene, auxiliary negative arm)
Opn4: melanopsin, ipRGC photopigment
Otp: orthopedia homeobox transcription factor
P(x): post-natal day x of development
Pacap: pituitary adenylate cyclase-activating peptide (ipRGC neuropeptide)
Per1/2: period 1/2 (clock gene, core negative arm)
Pou2f2: Pou class 2 homeobox 2 transcription factor
Prok2: prokineticin-2 (SCN neuropeptide)
Prokr2: prokineticin receptor 2
PVN: paraventricular nucleus of the hypothalamus
Rax: retinal and anterior neural fold homeobox transcription factor
RHT: retinohypothalamic tract
Ror/α/β: retinoic-acid-receptor related orphan nuclear receptor α/β (clock gene, auxiliary positive arm)
SCN: suprachiasmatic nucleus
Shh: Sonic hedgehog (lipid-modified polypeptide morphogen)
Sim1: single-minded homolog 1 basic-helix-loop-helix transcription factor
Six3/6: Six family homeodomain transcription factor 3/6
Sox2: sex-determining-region Y box 2 transcription factor
Sp: substance P, neuropeptide
SPZ: subparaventricular zone of the hypothalamus
vAH: ventral anterior hypothalamus (developmental compartment that gives rise to SCN and other nearby nuclei)
Vax1: ventral anterior homeobox 1
Vipr2: Vip receptor 2
Vip: vasoactive intestinal peptide (SCN neuropeptide)
vlSCN: ventrolateral core subdomain of the SCN, classically defined by Vip expression
Wnt: wingless family members (lipid-modified glycoprotein morphogens).
